# Levetiracetam Use in the Critical Care Setting

**DOI:** 10.3389/fneur.2013.00121

**Published:** 2013-08-23

**Authors:** Jennifer L. DeWolfe, Jerzy P. Szaflarski

**Affiliations:** ^1^Department of Neurology, UAB Epilepsy Center, University of Alabama at Birmingham (UAB), Birmingham, AL, USA

**Keywords:** intravenous levetiracetam, status epilepticus, pediatric population, stroke-related seizures, post-traumatic seizures, loading dose, therapeutic monitoring

## Abstract

Intravenous (IV) levetiracetam (LEV) is currently approved as an alternative or replacement therapy for patients unable to take the oral form of this antiepileptic drug (AED). The oral form has Food and Drug Administration (FDA) indications for adjunctive therapy in the treatment of partial onset epilepsy ages 1 month or more, myoclonic seizures associated with juvenile myoclonic epilepsy starting with the age of 12 and primary generalized tonic-clonic seizures in people 6 years and older. Since the initial introduction, oral and IV LEV has been evaluated in various studies conducted in the critical care setting for the treatment of status epilepticus, stroke-related seizures, seizures following subarachnoid or intracerebral hemorrhage, post-traumatic seizures, tumor-related seizures, and seizures in critically ill patients. Additionally, studies evaluating rapid infusion of IV LEV and therapeutic monitoring of serum LEV levels in different patient populations have been performed. In this review we present the current state of knowledge on LEV use in the critical care setting focusing on the IV uses and discuss future research needs.

## Introduction

Currently, intravenous (IV) levetiracetam (LEV) is approved only as an alternative or replacement therapy for patients unable to take the oral form of this antiepileptic drug (AED). The oral form of LEV is also approved for use in patients with multiple types of seizures and epilepsies. But, this AED has been increasingly used in the critical care setting (e.g., emergency rooms or intensive care units) due to its relative ease of use, positive outcomes, and the low side effects profile which are thought to be better than some of the other commonly used in this setting AEDs, e.g., phenytoin (PHT) (1, 2). When it was introduced to the market, LEV was marketed as an AED with a novel structure and mechanism of action – its main mechanism of action is modulating neurotransmitter release via binding to the synaptic vesicle protein 2A and, thus, via inhibiting calcium release from intracellular stores. Other mechanisms of action include opposition of the negative modulation of gammabutyric acid (GABA-) and glycine-gated currents, inhibition of the neuronal synchronization and of the N-type calcium channels (3). Oral LEV is rapidly and almost completely absorbed with plasma peak concentration reached within 1 h of intake but food can delay and reduce the peak concentration without an effect on bioavailability (4). In ICU patients who have received LEV for seizure prophylaxis (500 mg every 12 h) the clearance of LEV was faster when compared to the similar values obtain in healthy controls and patients in status epilepticus (SE); Monte Carlo simulation determined the most optimal LEV doses in these patients to achieve appropriate serum concentration should be either 1,000 mg every 8 h or 1,500–2,000 mg every 12 h (5). In patients with or without preexisting epilepsy who presented with SE and who were taking between none and several concomitant AEDs the pharmacokinetic data of IV infusion were comparable to the previously published values derived from healthy volunteers (6) while doses of IV LEV that were antiepileptogenic in animal models of epilepsy (55 mg/kg/day) administered to patients with traumatic brain injury (TBI) resulted in comparable pharmacokinetics (PK) in children, adults, and elderly with similar results observed between days 3 and 30 of treatment (delay in *T*_max_ in elderly was observed but this was of unclear clinical significance) (7). One study in patients with subarachnoid hemorrhage (SAH) compared the plasma concentrations of LEV while receiving IV or parenteral forms for seizure prevention – when switched to parenteral form the levels decreased to 70% of the IV levels but complications in response to this change were not observed (8). Finally, LEV is known to suppress seizures in the animal models of epilepsy and pretreatment with LEV can delay or altogether prevent the development of kindled seizures (9–12). Thus, the overall very favorable clinical and pharmacokinetic profiles make LEV a desirable treatment option for the use in the critical care setting. The goal for this invited commentary was to review the available literature focusing on the use of LEV in the critical care setting and to provide recommendations for future research.

## Materials and Methods

An extensive literature search was performed evaluating studies for IV LEV use in critical setting for the management of SE in adults, stroke-related seizures, TBI, SAH, intracranial hemorrhage, seizure prophylaxis in patients undergoing surgery for brain tumors and its use in neonates, and children and blood levels and therapeutic monitoring.

## Results

### Special patient populations

#### Status epilepticus

The initial reports of the use of LEV in the setting of SE utilized oral doses administered via feeding tube in patients ages 16–91 years. One study reported complete seizure control in all patients within 12–96 h of the initial LEV administration and the other study reported good outcomes in 11/13 episodes of SE (13, 14). Since then and since the IV form of LEV became available in 2006, this AED has been frequently favored in the critical care setting over other AEDs because of the simplicity of administration, linear PK, lack of significant cardiovascular side effects and lack of interactions with other medications (1, 3). This includes the use of IV LEV for the treatment of all types of seizures and SE. Four open-label prospective clinical studies evaluated use of IV LEV in adults with convulsive SE and found IV LEV to be effective in terminating SE with minimal side effects (6, 15–17). In the first study, Fattouch et al. used LEV as first-line therapy to demonstrate resolution or significant reduction in SE and seizures in 8/9 elderly patients who had no seizure recurrence within 24 h and who did not report any adverse events (16). The study by Misra et al. randomized 79 patients with seizures lasting>5 min to an initial therapy with IV LEV 20 mg/kg over 15 min or IV lorazepam (LZP) 0.1 mg/kg over 2–4 min with switch-over in case of lack of efficacy (17). This study demonstrated similar efficacy for the treatment of SE between LEV (29/38; 76.3%) vs. LZP (31/41; 75.6%); after switch-over 88.9% were controlled with LZP vs. 70% with LEV. The 24-h seizure-free rate was 23/29 for LEV and 21/31 for LZP. However, LZP patients experienced a higher need of artificial ventilation (17). In another study, Uges et al. determined that IV LEV added to standard SE treatment [IV clonazepam and/or rectal diazepam followed as needed by PHT or valproic acid (VPA)], was feasible and safe (6). Finally, in the study by Eue et al. 43 patients with SE were treated with IV LEV 1,000 or 2,000 mg after treatment with benzodiazepines was deemed to be ineffective. IV LEV was well tolerated and terminated SE in 19/43 patients; LEV was more effective in simple focal, complex focal, and myoclonic SE than in non-convulsive, subtle, or secondarily generalized SE (0/8) (15).

Several retrospective studies of IV LEV for the treatment of various forms of SE were conducted in 236 adults (18–22). For example, one study found that LEV was effective in controlling SE in 57.5% of patients with higher chance of seizure control if used as initial therapy or add-on to benzodiazepines (BZD; 78.5%) than as an add-on to treatment (BZD plus PHT, VPA, or both; 46.1%) (18). A study by Alvarez et al. compared benzodiazepines plus second-line treatment with PHT, VPA, or LEV to find LEV to be less effective in controlling SE than VPA at 51.7 vs. 74.6% but there were no differences in outcomes at discharge between the three groups (19). Another study by Möddel et al. found that IV LEV (bolus or continuous infusion) resolved refractory SE in 69% of 36 patients; higher incidence of failures was associated with doses>3,000 mg/day, lack of bolus, treatment initiated>48 h after diagnosis, non-convulsive SE with coma, periodic lateralized epileptiform transients, acute cerebral lesion, and intubation narcosis (21). Overall, these studies used variable doses of LEV between 1,000 and 9,000 mg/day with or without initial bolus. Of importance, these studies reported low numbers of patients with side effects due to LEV which typically included nausea and vomiting (20, 21), elevated liver enzymes (20), and transient thrombocytopenia (22). The reported mortality was 17% (responders 4%, non-responders 45%) (21). The message from these prospective and retrospective data collections appears to be fairly clear – the efficacy of IV LEV for the management of SE appears to be between 48 and 94% (probably closer to 50%) with better efficacy reported with early LEV initiation and with pretreatment with BDZ as seen in studies of other AEDs in animal and human SE (23, 24).

#### Stroke-related seizures

The American Stroke Association’s guidelines for early management of adults with ischemic stroke state that prophylactic use of AEDs in patients with stroke who have not had seizures is not recommended (Class III, Level of Evidence C); but, if seizures after stroke occur, treatment should follow the guidelines for the management of seizures in other neurological conditions (Class I, Level of Evidence B) (25). Overall, three studies reported on treating 98 patients with post-stroke seizures with LEV (ages 57–89 years) (26–28). In one prospective study, 82.4% of patients were seizure-free on LEV doses ranging from 1,000 to 2,000 mg/day (27). Another study, reported on the treatment of early and late seizures in the setting of ischemic or hemorrhagic stroke and found that in ∼90% of patients seizures were controlled (26/29) with LEV dosed at 1,000–2,000 mg/day (28). Finally, Belcastro et al. treated 35 post-stroke seizure patients with LEV to report seizure freedom of 77.1% (26). Additional retrospective studies evaluated the efficacy of LEV in a total of 92 patients with early or late post-stroke seizures in doses of up to 3,000 mg/day (29–31). In either monotherapy or adjunctive therapy, in the majority of patients seizures were controlled. While the incidence of early and late seizures in patients with stroke (ischemic or hemorrhagic) is fairly high, reaching in some studies 10% or more (32, 33), and many calls made for the development of randomized controlled trials for seizure treatment or seizure prevention in these patients, such studies have not been conducted to date (34).

#### Post-traumatic seizures

According to the published guidelines, the prophylactic use of PHT may reduce early post-traumatic seizures (within 7 days; Class I) but this or other AEDs are not recommended for preventing late post-traumatic seizures (>7 days of injury; Class I) (35, 36). One open-label, non-randomized phase II study compared prophylactic LEV for 30 days (*N* = 66) to no AED use (observation; *N* = 60) in 86 adults and 40 children following TBI (37). Patients with early presentation (within 8 h of TBI) received LEV while patients presenting>8 h after TBI did not receive LEV. The severity of TBI was higher in the LEV-treated group (*p* = 0.03). This study reported seizure incidence of 10.9% in the treated group (more severe TBI group) vs. 20% in the observation group at 2 years but the difference was not significant (*p* = 0.18) (37). Two-year follow-up of the pediatric group (*N* = 40) revealed that only one patient developed late seizures/epilepsy (defined as seizures after the initial 7 days period) (38).

Several prospective studies of seizure prevention in adults following TBI focused on the use of LEV up to the dose of 4,000 mg/day. Szaflarski et al. in a prospective, single-blinded, randomized clinical trial compared LEV to PHT within 24 h of TBI or SAH in 52 patients (39). While there were no differences in seizure or mortality outcomes between the groups, patients dosed with LEV had better outcomes including lower Disability Rating Scale (DRS) scores at 3 months and higher Glasgow Outcomes Scale at 6 months when compared to patients treated with PHT. In this study, seizure incidence was based on the results of video/EEG monitoring conducted for up to 72 h after the initial dose of AED was administered which is considered standard in the setting of severe TBI (40). In another prospective non-randomized and not blinded study, Inaba et al. evaluated 813 patients with blunt TBI who were treated prophylactically with LEV (*N* = 406) or PHT (*N* = 407) and then monitored for the development for clinical seizures (no EEG monitoring) within 7 days (41). Further, patient distribution in the treatment arms was unbalanced with each center following local practice patterns and one of the centers preferentially utilizing LEV and the other PHT. Results demonstrated no differences in mortality (5.4 vs. 3.7%, *p* = 0.236), seizure rate (1.5 vs. 1.5%, *p* = 0.997), or adverse drug reactions (7.9 vs. 10.3%, p = 0.227) between the two groups. Jones et al. prospectively evaluated 32 patients who had received LEV for seizure prevention in the setting of severe TBI and compared them to 41 patients treated with PHT (42). While only some patients in each group received EEG, increased “seizure tendency” on EEG was observed in patients who have received LEV when compared to PHT (*p* = 0.003); seizure incidence between groups was similar (*p* = 0.556). In another report, 6/7 patients with post-traumatic epilepsy became seizure-free after initiation of add-on therapy with LEV but only a relatively short (10–16 months) follow-up period was reported (43). Adverse outcomes reported in these studies included headache, somnolence, memory impairment, irritability, dizziness, depression, and ataxia with some of the studies reporting higher incidence of adverse outcomes in patients receiving PHT (39, 41, 42).

Approximately 30% of the use of LEV in the critical care setting is for seizure prophylaxis in patients with TBI (1) but the data to support such use are incomplete. Randomized and double-blinded studies are needed to address this unmet need and to provide unambiguous data regarding the short- and long-term outcomes (seizures/epilepsy, cognitive, quality of life, etc.) in patients with TBI.

#### Seizures following subarachnoid or intracerebral hemorrhage

The published guidelines recommend prophylactic anticonvulsant use in the immediate post-hemorrhagic period in patients with aneurysmal SAH (Class IIb, Level of Evidence B) but discourage routine long-term use of anticonvulsants (Class III, Level of Evidence B) (44, 45). Prospective studies in this population include one that compared IV LEV (*N* = 18) to IV VPA (*N* = 17) and demonstrated no difference in seizure occurrence between the groups and no adverse effects in the group using LEV (8). In a convenience sample of 442 consecutive patients with SAH (*N* = 297 treated before ICU protocol change with IV PHT load followed by 14 days of PHT treatment with doses adjusted based on the presence of low levels or seizures and *N* = 145 treated with IV LEV 500 mg twice daily without loading dose for 3 days after protocol change) Murphy-Human et al. found no difference in early seizures, mortality rate, and intensive care unit or total hospital stay in patients with SAH. There was an increased likelihood of late seizures (≥3 days post-SAH) and in-hospital seizures in the LEV group. However, the significant differences in treatment pattern between AEDs in this study (lack of loading and much shorter treatment with LEV) make the comparison of efficacy for seizure prevention between the groups difficult which the authors recognize as a shortcoming (46). A prospective observational study in patients with intracerebral hemorrhage (ICH) found similar risk of seizures between patients who had received PHT (*N* = 28) and LEV (*N* = 18) for seizure prevention (*p* > 0.1) but patients treated with PHT fared overall worse with increased risk of poor outcome (*p* = 0.02) and more adverse events of treatment; (47) these results have confirmed their previous findings of poorer outcomes in patients with SAH treated for seizure prophylaxis with PHT (48).

A retrospective study of the prophylactic use of PHT (*N* = 25; loading dose 15–20 mg/kg with later adjustments of the dose) or LEV (*N* = 60; dose 500–2,000 mg/day) in patients with ICH (*N* = 40), SAH (*N* = 26) or subdural hemorrhage (SDH; *N* = 19) found patients treated with LEV to have higher Glasgow Coma Scale (GCS) scores at discharge, lower seizure incidence, and higher percentage discharge home when compared to the PHT group (2). Trend toward better cognitive outcomes in the LEV group was also observed (*p* = 0.08). Shah and Husain retrospectively evaluated 176 patients with post-aneurysmal SAH who received prophylactic treatment with PHT (loading dose 20 mg/kg and maintenance dose 5–7 mg/kg/day) who were later transitioned to LEV (1,500 mg twice daily) due to adverse events including elevated transaminases, thrombocytopenia, rash, unexplained fever, mental status decline, or gastrointestinal (GI) disturbance; all but one patient switched to LEV with GI disturbance and three patients with mental status abnormalities had subsequent improvement or resolution of symptoms at discharge or by the first follow-up visit (14–41 days following discharge). Adverse events occurred more frequently in the PHT group and there were no clinical seizures in the LEV group (49).

#### Tumor-related seizures

It should not be a surprise to note LEV being used in the setting of seizure prevention or seizure treatment in patients with central nervous system (CNS) malignancies – several early studies reported positive experiences in this setting (50, 51). The main reason for this switch in practice pattern is the fact that the newer AEDs (including LEV) do not interfere with the metabolism of chemotherapeutics and, thus, do not negatively affect their efficacy (52). Overall, seizures/epilepsy is common in patients with brain malignancies ranging from ∼10% in patients with CNS lymphomas and up to 100% in dysembryoplastic tumors (53). Generally, initiation of therapy with an AED is warranted in patients who had at least one seizure in the setting of a brain tumor but whether an AED should be initiated in patients with brain tumors who have not experienced a seizure is less clear. Depending on type of tumor, age, location, etc., patients diagnosed with CNS malignancies have 20–45% chance of developing seizures (53). Some authorities suggest the use of LEV or gabapentin as first-line therapy for the treatment of seizures in patients with brain tumors (54). One of the first LEV studies in this population enrolled 26 patients with gliomas – LEV was used as an add-on therapy from 2,000 to 4,000 mg/day to achieve seizure reduction of>50% in 65% of the patients (4/20 previously refractory patients became seizure-free) (51). A prospective observational study enrolled 30 patients with brain tumors and epilepsy who were treated with LEV administered for 4 weeks prior to and for 4 weeks following respective procedure (*N* = 25 for the post-surgical group) (55). Initial doses were 1,000 mg/day with dose escalation in case of seizures up to 3,000 mg/day. Of the 25 patients, 88% were seizure-free at 48 h and 84% were seizure-free at 4 weeks following surgery (55). Another prospective open-label study evaluated treatment with LEV monotherapy for the first post-resection month in 17 patients with brain tumors who had>1 seizure within 1 month prior to surgery (56). Postoperatively, all patients received IV LEV for 48 h at 500 mg BID or pre-surgery dose, then titrated up by 500 mg/day to goal 3,000 mg/day as tolerated. There was a>50% reduction in seizures in 11/12 patients who completed the study. Lim et al. conducted a prospective, open-label study of transition from monotherapy with PHT to monotherapy with LEV in 29 patients for postoperative control of glioma-related seizures (1/3 continued on PHT while 2/3 transitioned to LEV) (57). At 6 months after surgery, 87% (13/15) of patients on LEV and 75% (6/8) of patients on PHT were seizure-free. Both groups had similar incidence of excessive sleepiness, sleeping difficulty, and lack of energy or strength, although increased incoordination in PHT group and increased slurred speech in LEV group.

Finally, Milligan and colleagues performed a retrospective analysis on the incidence of early seizures and postoperative epilepsy in 315 adults following supratentorial surgery who received prophylactic monotherapy LEV (500–3,000 mg/day) vs. monotherapy PHT (200–800 mg/day). Ninety-nine patients had a primary brain tumor and in those patients, early seizures occurred in 2.3% on LEV and 3.6% on PHT. Fifty-five of the 99 patients were followed>12 months and 5/11 on LEV and 24/44 on PHT developed epilepsy. Thirty-eight patients on PHT vs. one patient on LEV discontinued AED treatment due to side effects (*p* = 0.03) (58). Another retrospective study evaluated prophylactic use of LEV (1,000–3,000 mg/day) in 78 patients with supratentorial brain tumors. Preoperative seizure incidence was 38.5% and postoperative seizures occurred in 2.6% (2/78) patients with 91% of patients being seizure-free at the end of the mean follow-up to 10.5 months (59). Finally, Hildebrand et al. reported on the use of various AEDs in the setting of brain tumors including LEV to find epilepsy in 80% of their patients; the typical dose was 1,000–3,000 mg/day but the treatment of LEV was not compared specifically to other AEDs (60).

While substantial body of evidence is available regarding the treatment of seizures in the setting of brain tumors or supratentorial surgery for the management of brain tumors and some have advocated the use of LEV in this setting after the data by Milligan et al. were published (58, 61), careful prospective studies are needed to assess the use of LEV as a preventive AED in this setting, to evaluate complex interaction between surgery, chemotherapy, and AEDs and, finally, whether LEV should be the preferred AED in this setting instead of PHT or VPA (53, 54).

#### Geriatric population

There were no observed safety differences between 347 geriatric patients (age ≥ 65) and younger patients treated with LEV for seizures, although the number of the elderly patients enrolled in the controlled trials of epilepsy is insufficient to determine the effectiveness of LEV in this population [package insert (62)]. Nevertheless, geriatric patients have been enrolled in many of the retrospective and prospective studies of LEV including studies that used IV doses of LEV. For example, Uges et al. analyzed safety and PK of IV infusion of LEV in patients with SE ages 44–75 years of age (median 60 years) to show PK values in the studied group similar to norms obtained from healthy (and younger) volunteers (6). Another study by Klein et al. showed that *T*_max_ was longer in subjects older than 65 years of age when compared to children and young adults at the initiation of the therapy and at 30 days (7). In the elderly LEV appears to be safe and associated with a relatively low level of adverse events. In part, this is related to lack of significant drug–drug interactions. Overall, PK studies and safety/efficacy studies of LEV in the elderly are needed as the incidence and prevalence of epilepsy, and thus the use of AEDs in this population are increasing.

#### The use of IV LEV in the pediatric population

When initially approved by the FDA, IV LEV was not indicated for use in children less than 16 years of age. Since then, prospective studies using IV LEV to treat acute seizures in neonates and children have assessed the safety and efficacy of LEV use in these age groups (63, 64). Ramantani et al. conducted a prospective feasibility study in 38 newborns with LEV applied as first-line treatment for EEG-confirmed seizures (64). In this study the initial IV dose was 10 mg/kg with gradual increase up to 45–60 mg/kg over 7 days; 30/38 infants were seizure-free at the end of the evaluation period (22 had to receive additional doses of phenobarbital). Another study evaluated a single dose of IV LEV 50 mg/kg infused over 15 min in 30 children (mean age 6.3 years; range 6 months to 14.8 years) diagnosed with epilepsy (29/30) or a single seizure related to a brain lesion. The mean blood level 10 min after infusion was 83.3 mcg/mL (47–128 mcg/mL); administration of LEV was associated with a subsequent reduction of all seizure types for up to 24-h after the infusion (63). Adverse events in both studies included sleepiness and/or fatigue, drowsiness with titration, and thrombocytopenia with concurrent VPA use. Further studies utilizing IV LEV for the treatment of acute seizures in 189 pediatric patients (1 day to 18 years) in eight retrospective case series and two case reports resulted an improved clinical seizure control; 118/189 reported concurrent EEG monitoring which demonstrated improved electrographic seizure control (65–74).

### Methods of infusion and monitoring

#### Rapid infusion

Intravenous LEV is supplied in a concentrated form that needs to be diluted in compatible diluent prior to administration. While the IV formulation is reported to be bioequivalent to the oral formulation and doses should be interchangeable some differences in bioavailability between the IV and parenteral doses have been reported (8). There are also some age-related differences in PK (7) but it is unclear whether these differences are of clinical significance. In one study, Wheless et al. assessed rapid infusion (over 5–6 min) of 20, 40, and 60 mg/kg (*N* = 15 per group) of IV LEV in children and adults (4–32 years of age). Maximum plasma concentration peaked 15 min after infusion. The infusion was well tolerated with minimal side effects including non-pruritic rash (*N* = 1) and infusion site pain (*N* = 2); there were no electrocardiographic changes reported (75).

#### Therapeutic monitoring of serum LEV levels in different patient populations

Although therapeutic serum concentration ranges and a schedule for blood level monitoring for LEV have not been established, monitoring is recommended, e.g., from pregnancy through the postpartum period due to physiologic changes leading to gradual decreases in LEV plasma levels with the advancement of the pregnancy (62). One prospective study in 30 epilepsy patients on>2 AEDs, including LEV in doses ranging from 2,000 to 3,000 mg/day, defined the therapeutic LEV plasma range of 10–40 mcg/mL (76). Eighteen patients were either seizure-free (*N* = 5) or had>50% seizure reduction (*N* = 13). The majority of patients had an associated therapeutic LEV range in the low-therapeutic range. In patients with LEV level within the low-therapeutic range adjustments in dose produced either further therapeutic response or allowed for the patients to be weaned from one of the other AEDs without any ill effects. Another study in 297 inpatients using LEV in doses 250–7,000 mg/day demonstrated serum concentrations 1.5–48.2 mcg/mL with the level to dose ratio (LDR) lower in LEV monotherapy compared to concurrent use of enzyme inducing AEDs (77). In this study, the median LDR was significantly lower when patients were co-medicated with enzyme inducer (e.g., PHT, carbamazepine, or oxcarbazepine) when compared to LEV monotherapy whereas the LDR of patients co-medicated with VPA or lamotrigine did not differ significantly from the LDR of LEV of patients on LEV monotherapy (*p* > 0.05); children had lower LEV concentrations than adults on the same dose per body weight (77).

In a pooled analysis of LEV levels in 1,023 patients enrolled in four Phase III double-blind trials and during which patients receiving one to three concomitant AEDs were treated with LEV (*N* = 672) or placebo (*N* = 351) as adjunctive therapy to treat seizures (78–81), LEV concentrations were normalized to a dose of 1 mg/kg twice daily with mean plasma concentration at 1 h ranging between 1.74 and 2.27 μg/mL at 1,000–4,000 mg/day and a mean plasma LEV level concentration of 2.09 μg/mL (95% CI 1.99, 2.19) (82). The mean plasma LEV concentration at 12 h ranged from 0.7 to 0.88 μg/mL at 1,000–4,000 mg/day and a mean plasma LEV level concentration of 0.82 μg/mL (95% CI 0.19, 0.85). LEV concentrations were lower (<25% on average) in patients using concurrent enzyme inducing AEDs and moderately higher in patients using concurrent VPA (12 h post-dose). Two retrospective studies in 73 adults with epilepsy reported LEV doses ranging from 1,000 to 4,000 mg/day with therapeutic plasma concentrations between 6 and 65 μg/mL (83, 84). Adverse events leading to LEV discontinuation included behavioral changes (*N* = 3), gait disturbance (*N* = 1), and depression (*N* = 1) (83, 84). Two retrospective studies in pediatric patients reported LEV doses in 93 children ranging from 12.7 to 84 mg/kg/day with blood levels in responders ranging from 5 to 60 μg/mL (85, 86). None of these pediatric and adult studies reported dose – level – seizure response relationship.

#### Neurocritical care patients

In a prospective open-label, steady-state pharmacokinetic study 12 adults admitted to the neurocritical care unit with SAH, SDH, or TBI were treated prophylactically with IV LEV (5). Doses of 1,000 mg every 8 h and 1500–2000 mg every 12 h were most likely to achieve trough levels between 6 and 20 μg/mL than doses of 500 mg twice daily; these critically ill patients demonstrated faster systemic clearance and shorter terminal elimination half-life compared to previously published data on healthy volunteers and adults in SE. Another prospective single-center registry in 35 critically ill patients with aneurysmal SAH reported decreased LEV plasma concentrations after transition from IV to parenteral dose with concurrent decrease in bioavailability by ∼30% (8).

## Summary

Intravenous LEV is a safe and effective treatment for acute seizures and SE and has fewer side effects than some traditional first-line agents. The evidence suggests that early treatment and use in focal and myoclonic SE may be more effective than in secondarily generalized SE. However, large controlled and blinded studies are needed to answer these questions. Most studies in patients with SAH or ICH demonstrated no difference in early seizures or mortality with prophylactic use of LEV when compared to other AEDs. Only one prospective study suggested increased rate of late seizures in patients on LEV compared to PHT, however IV formulation was changed to enteral formulation and it is unclear how that may have affected the outcome; the treatment with LEV in this study was overall shorter and the LEV dose substantially lower than the dose of the comparator – PHT which may have affected the results. Several studies in patients with SAH, ICH, or TBI found decreased side effects in patients on LEV vs. PHT or VPA. There was no difference in rate of post-traumatic seizures or mortality in patients with TBI whether treated with LEV or PHT, however reduced disability scores at 3 months and higher Glasgow Outcomes Scale scores at 6 months in patients on LEV suggest a potential neuroprotective effect of LEV which is in agreement with animal studies. IV LEV has proven to be effective and safe for use in treating acute seizures in children of all ages from premature neonates to teenagers. Rapid infusion of IV LEV over 5 min in children and adults is safe and well tolerated. Therapeutic LEV monitoring is important to perform in some patient populations, especially in those who are critically ill, but the relationship between the dose – level – seizure response has not been established. Neurocritical care patients may have increased clearance with a shorter half-life compared to patients who are healthy or in SE patients.

## Future Directions

The above presented data collected prospectively or retrospectively support further studies of the use of LEV in the setting of CNS emergencies whether for seizure prevention/treatment or for assessing the short- and long-term cognitive and societal outcomes (e.g., employment, quality of life, etc.). Further, randomized and double-blind studies of acute seizures and SE across ages appear to be warranted. Long-term neurological functional and disability status outcomes after administering IV LEV within 24 h of TBI should be performed to confirm the neuroprotective effects of LEV observed in animal studies. Further studies should be performed to evaluate effective doses of LEV in critically ill patients and determine optimal schedule for therapeutic monitoring. Although some studies suggest IV LEV is safe and tolerable in geriatric patients, larger prospective studies are needed to determine the efficacy in this population, including potentially decreased renal function.

## Key Concepts

1. Intravenous LEV is effective in terminating many types of seizures and SE, including convulsive SE and partial SE and is well tolerated with minimal side effects unlike some typical first and second-line agents.
2. In patients with TBI or intracranial hemorrhage long-term prophylaxis with LEV vs. PHT may not alter the incidence of seizures or mortality, however, patients treated with LEV may have better long-term outcomes.
3. In prospective and retrospective studies IV LEV appears to be a safe and effective treatment for acute seizures in premature and term newborns, school-aged children, and teenagers.
4. IV LEV can be infused rapidly over 5 min with maximal peak concentration in 15 min without significant clinical or electrocardiographic side effects in children and adults.
5. Critical care patients may have faster systemic clearance and shorter terminal elimination half-life of IV LEV compared to previously published data on healthy volunteers and adults in SE. Conversion to enteral formulation in these patients may result in lower plasma concentrations. Serum level monitoring should be considered in critically ill patients though the clinical importance of the monitoring is not clear.

## Author Contributions

Jennifer L. DeWolfe and Jerzy P. Szaflarski are original authors of this manuscript.

## Conflict of Interest Statement

Jennifer L. DeWolfe performs or has performed research sponsored by NIH, UCB Pharma, Eiasi, and GlaxoSmithKline. Jerzy P. Szaflarski performs or has performed research sponsored by NIH, FDA, Shor Foundation for Epilepsy Research, and UCB Pharma.

## References

[1] Szaflarski JP Meckler JM Szaflarski M Shutter LA Privitera MD Yates SL Levetiracetam use in critically ill patients. Neurocrit Care (2007) 7:140–7 10.1007/s12028-007-0042-8 17607530

[2] Taylor S Heinrichs RJ Janzen JM Ehtisham A Levetiracetam is associated with improved cognitive outcome for patients with intracranial hemorrhage. Neurocrit Care (2011) 15:80–4 10.1007/s12028-010-9341-6 20890680

[3] Abou-Khalil B Levetiracetam in the treatment of epilepsy. Neuropsychiatr Dis Treat (2008) 4:507–23 10.2147/NDT.S2937 18830435 PMC2526377

[4] Patsalos PN The pharmacokinetic characteristics of levetiracetam. Methods Find Exp Clin Pharmacol (2003) 25:123–9 10.1358/mf.2003.25.2.723686 12731458

[5] Spencer DD Jacobi J Juenke JM Fleck JD Kays MB Steady-state pharmacokinetics of intravenous levetiracetam in neurocritical care patients. Pharmacotherapy (2011) 31:934–41 10.1592/phco.31.10.934 21950640

[6] Uges JW van Huizen MD Engelsman J Wilms EB Touw DJ Peeters E Safety and pharmacokinetics of intravenous levetiracetam infusion as add-on in status epilepticus. Epilepsia (2009) 50:415–21 10.1111/j.1528-1167.2008.01889.x 19054418

[7] Klein P Herr D Pearl PL Natale J Levine Z Nogay C Results of phase II pharmacokinetic study of levetiracetam for prevention of post-traumatic epilepsy. Epilepsy Behav (2012) 24:457–61 10.1016/j.yebeh.2012.05.011 22771222 PMC4561854

[8] Mink S Muroi C Seule M Bjeljac M Keller E Levetiracetam compared to valproic acid: plasma concentration levels, adverse effects and interactions in aneurysmal subarachnoid hemorrhage. Clin Neurol Neurosurg (2011) 113:644–8 10.1016/j.clineuro.2011.05.007 21703756

[9] Klitgaard H Matagne A Gobert J Wulfert E Evidence for a unique profile of levetiracetam in rodent models of seizures and epilepsy. Eur J Pharmacol (1998) 353:191–206 10.1016/S0014-2999(98)00410-5 9726649

[10] Loscher W Honack D Rundfeldt C Antiepileptogenic effects of the novel anticonvulsant levetiracetam (ucb L059) in the kindling model of temporal lobe epilepsy. J Pharmacol Exp Ther (1998) 284:474–9 9454787

[11] Loscher W Reissmuller E Ebert U Anticonvulsant efficacy of gabapentin and levetiracetam in phenytoin-resistant kindled rats. Epilepsy Res (2000) 40:63–77 10.1016/S0920-1211(00)00108-X 10771259

[12] Stratton SC Large CH Cox B Davies G Hagan RM Effects of lamotrigine and levetiracetam on seizure development in a rat amygdala kindling model. Epilepsy Res (2003) 53:95–106 10.1016/S0920-1211(02)00254-1 12576171

[13] Patel NC Landan IR Levin J Szaflarski J Wilner AN The use of levetiracetam in refractory status epilepticus. Seizure (2006) 15:137–41 10.1016/j.seizure.2005.12.003 16426869

[14] Rossetti AO Bromfield EB Levetiracetam in the treatment of status epilepticus in adults: a study of 13 episodes. Eur Neurol (2005) 54:34–8 10.1159/000087385 16103676

[15] Eue S Grumbt M Muller M Schulze A Two years of experience in the treatment of status epilepticus with intravenous levetiracetam. Epilepsy Behav (2009) 15:467–9 10.1016/j.yebeh.2009.05.020 19616482

[16] Fattouch J Di Bonaventura C Casciato S Bonini F Petrucci S Lapenta L Intravenous levetiracetam as first-line treatment of status epilepticus in the elderly. Acta Neurol Scand (2010) 121:418–21 10.1111/j.1600-0404.2010.01351.x 20578996

[17] Misra UK Kalita J Maurya PK Levetiracetam versus lorazepam in status epilepticus: a randomized, open labeled pilot study. J Neurol (2012) 259:645–8 10.1007/s00415-011-6227-2 21898137

[18] Aiguabella M Falip M Villanueva V de laPeña P Molins A Garcia-Morales I Efficacy of intravenous levetiracetam as an add-on treatment in status epilepticus: a multicentric observational study. Seizure (2011) 20:60–4 10.1016/j.seizure.2010.10.009 21145758

[19] Alvarez V Januel JM Burnand B Rossetti AO Second-line status epilepticus treatment: comparison of phenytoin, valproate, and levetiracetam. Epilepsia (2011) 52:1292–6 10.1111/j.1528-1167.2011.03056.x 21480881

[20] Berning S Boesebeck F van Baalen A Kellinghaus C Intravenous levetiracetam as treatment for status epilepticus. J Neurol (2009) 256:1634–42 10.1007/s00415-009-5166-7 19458986

[21] Möddel G Bunten S Dobis C Kovac S Dogan M Fischera M Intravenous levetiracetam: a new treatment alternative for refractory status epilepticus. J Neurol Neurosurg Psychiatry (2009) 80:689–92 10.1136/jnnp.2008.145458 19448097

[22] Ruegg S Naegelin Y Hardmeier M Winkler DT Marsch S Fuhr P Intravenous levetiracetam: treatment experience with the first 50 critically ill patients. Epilepsy Behav (2008) 12:477–80 10.1016/j.yebeh.2008.01.004 18291724

[23] Borris DJ Bertram EH Kapur J Ketamine controls prolonged status epilepticus. Epilepsy Res (2000) 42:117–22 10.1016/S0920-1211(00)00175-3 11074184 PMC2885610

[24] Gaspard N Foreman B Judd LM Brenton JN Nathan BR McCoy BM Intravenous ketamine for the treatment of refractory status epilepticus: a retrospective multicenter study. Epilepsia (2013) 54(8):1498–503 10.1111/epi.12247 23758557 PMC3731413

[25] Adams HP Jr del Zoppo G Alberts MJ Bhatt DL Brass L Furlan A Guidelines for the early management of adults with ischemic stroke: a guideline from the American Heart Association/American Stroke Association Stroke Council, Clinical Cardiology Council, Cardiovascular Radiology and Intervention Council, and the Atherosclerotic Peripheral Vascular Disease and Quality of Care Outcomes in Research Interdisciplinary Working Groups: the American Academy of Neurology affirms the value of this guideline as an educational tool for neurologists. Stroke (2007) 38:1655–711 17431204 10.1161/STROKEAHA.107.181486

[26] Belcastro V Costa C Galletti F Autuori A Pierguidi L Pisani F Levetiracetam in newly diagnosed late-onset post-stroke seizures: a prospective observational study. Epilepsy Res (2008) 82:223–6 10.1016/j.eplepsyres.2008.08.008 18829259

[27] Kutlu G Gomceli YB Unal Y Inan LE Levetiracetam monotherapy for late poststroke seizures in the elderly. Epilepsy Behav (2008) 13:542–4 10.1016/j.yebeh.2008.04.025 18539085

[28] Natale E Vinci G Ornella D Ronnasa T Effects of levetiracetam on stroke-related seizures: an open-label trial: preliminary report. In: Annual Meeting of the American Epilepsy Society. New Orleans: Blackwell Publishing (2004). 310 p

[29] Falip M Carreño M Amaro S Donaire A Delgado R Toledo M Use of levetiracetam in hospitalized patients. Epilepsia (2006) 47:2186–8 10.1111/j.1528-1167.2006.00850.x 17201722

[30] Feleppa M Fucci S Di Iorio W Apice G D’Aarenio M Treatment of early and late post-stroke seizures with levetiracetam in monotherapy. A safe and effective therapeutic option in preventing recurrent seizures [abstract]. Epilepsia (2009) 50:5019298432

[31] Sachdev H Dham G Velpuri A Foo E Levetiracetam in poststroke seizures in a community hospital [abstract]. Epilepsia (2004) 45:128

[32] Szaflarski JP Rackley AY Kleindorfer DO Khoury J Woo D Miller R Incidence of seizures in the acute phase of stroke: a population-based study. Epilepsia (2008) 49:974–81 10.1111/j.1528-1167.2007.01513.x 18248443 PMC5316476

[33] Vespa PM O’Phelan K Shah M Mirabelli J Starkman S Kidwell C Acute seizures after intracerebral hemorrhage: a factor in progressive midline shift and outcome. Neurology (2003) 60:1441–6 10.1212/01.WNL.0000063316.47591.B4 12743228

[34] Herman ST Early poststroke seizures: is it time for prospective treatment trials? Neurology (2011) 77:1776–8 10.1212/WNL.0b013e31823b4e73 22013179

[35] Chang B Lowenstein D Practice parameter: antiepileptic drug prophylaxis in severe traumatic brain injury. Neurology (2003) 60:10–6 10.1212/01.WNL.0000031432.05543.14 12525711

[36] Foundation BT Guidelines of the management of severe traumatic brain injury 3rd edition: XII. Seizure prophylaxis. J Neurotrauma (2007) 24:83–6

[37] Klein P Herr D Pearl PL Natale J Levine Z Nogay C Results of phase 2 safety and feasibility study of treatment with levetiracetam for prevention of posttraumatic epilepsy. Arch Neurol (2012) 69:1290–5 22777131 10.1001/archneurol.2012.445 PMC4798941

[38] Pearl PL McCarter R McGavin CL Yu Y Sandoval F Trzcinski S Results of phase II levetiracetam trial following acute head injury in children at risk for posttraumatic epilepsy. Epilepsia (2013). 10.1111/epi.12326 23876024 PMC3769484

[39] Szaflarski JP Sangha KS Lindsell CJ Shutter LA Prospective, randomized, single-blinded comparative trial of intravenous levetiracetam versus phenytoin for seizure prophylaxis. Neurocrit Care (2010) 12:165–72 10.1007/s12028-009-9304-y 19898966

[40] Steinbaugh LA Lindsell CJ Shutter LA Szaflarski JP Initial EEG predicts outcomes in a trial of levetiracetam vs. fosphenytoin for seizure prevention. Epilepsy Behav (2012) 23:280–4 10.1016/j.yebeh.2011.12.005 22342434

[41] Inaba K Menaker J Branco BC Gooch J Okoye OT Herrold J A prospective multicenter comparison of levetiracetam versus phenytoin for early posttraumatic seizure prophylaxis. J Trauma Acute Care Surg (2013) 74:766–73 10.1097/TA.0b013e3182826e84 23425733

[42] Jones KE Puccio AM Harshman KJ Falcione B Benedict N Jankowitz BT Levetiracetam versus phenytoin for seizure prophylaxis in severe traumatic brain injury. Neurosurg Focus (2008) 25:E3 10.3171/FOC.2008.25.10.E3 18828701 PMC3705765

[43] McCabe P McNew C Michel N Patients with intractable epilepsy secondary to head trauma with abnormal imaging studies: high response rate with add-on levetiracetam. In: Annual Meeting of the American Epilepsy Society. New Orleans: Blackwell Science, Inc (2004). [*Epilepsia* (Suppl 7):264].

[44] Connolly ES Jr Rabinstein AA Carhuapoma JR Derdeyn CP Dion J Higarshida RT Guidelines for the management of aneurysmal subarachnoid hemorrhage: a guideline for healthcare professionals from the American Heart Association/American Stroke Association. Stroke (2012) 43:1711–37 10.1161/STR.0b013e3182587839 22556195

[45] Society NC Critical care management of patients following aneurysmal subarachnoid hemorrhage: recommendations from the neurocritical care society’s multidisciplinary consensus conference. Neruocrit Care (2011) 15:211–40 10.1007/s12028-011-9605-9 21773873

[46] Murphy-Human T Welch E Zipfel G Diringer MN Dhar R Comparison of short-duration levetiracetam with extended-course phenytoin for seizure prophylaxis after subarachnoid hemorrhage. World Neurosurg (2011) 75:269–74 10.1016/j.wneu.2010.09.002 21492729

[47] Naidech AM Garg RK Liebling S Levasseur K Macken MP Schuele SU Anticonvulsant use and outcomes after intracerebral hemorrhage. Stroke (2009) 40:3810–5 10.1161/STROKEAHA.109.559948 19797183

[48] Naidech AM Kreiter KT Janjua N Ostapkovich N Parra A Commichau C Phenytoin exposure is associated with functional and cognitive disability after subarachnoid hemorrhage. Stroke (2005) 36:583–7 10.1161/01.STR.0000141936.36596.1e 15662039

[49] Shah D Husain AM Utility of levetiracetam in patients with subarachnoid hemorrhage. Seizure (2009) 18:676–9 10.1016/j.seizure.2009.09.003 19864168

[50] Ficker D Meckler J Szaflarski J Shutter L Warnick R Use of Levetiracetam as pre-operative prophylaxis in brain tumour surgery patients. In: 26th International Epilepsy Congress Paris. Paris: Blackwell Science, Inc (2005). [*Epilepsia* (Suppl 6):106].

[51] Wagner GL Wilms EB Van Donselaar CA Vecht ChJ Levetiracetam: preliminary experience in patients with primary brain tumours. Seizure (2003) 12:585–6 10.1016/S1059-1311(03)00096-7 14630498

[52] Relling MV Pui CH Sandlund JT Rivera GK Hancock ML Boyett JM Adverse effect of anticonvulsants on efficacy of chemotherapy for acute lymphoblastic leukaemia. Lancet (2000) 356:285–90 10.1016/S0140-6736(00)02503-4 11071183

[53] van Breemen MS Wilms EB Vecht CJ Epilepsy in patients with brain tumours: epidemiology, mechanisms, and management. Lancet Neurol (2007) 6:421–30 10.1016/S1474-4422(07)70103-5 17434097

[54] van Breemen MS Vecht CJ Optimal seizure management in brain tumor patients. Curr Neurol Neurosci Rep (2005) 5:207–13 10.1007/s11910-005-0048-6 15865886

[55] Bähr O Hermisson M Rona S Rieger J Nussbaum S Körtvelyessy P Intravenous and oral levetiracetam in patients with a suspected primary brain tumor and symptomatic seizures undergoing neurosurgery: the HELLO trial. Acta Neurochir (2012) 154:229–35 discussion 35, 10.1007/s00701-011-1144-9 21909835

[56] Usery JB Michael LM II Sills AK Finch CKA prospective evaluation and literature review of levetiracetam use in patients with brain tumors and seizures. J Neurooncol (2010) 99:251–60 10.1007/s11060-010-0126-8 20146087

[57] Lim DA Tarapore P Chang E Burt M Chakalian L Barbaro N Safety and feasibility of switching from phenytoin to levetiracetam monotherapy for glioma-related seizure control following craniotomy: a randomized phase II pilot study. J Neurooncol (2009) 93:349–54 10.1007/s11060-008-9781-4 19169651 PMC2687520

[58] Milligan TA Hurwitz S Bromfield EB Efficacy and tolerability of levetiracetam versus phenytoin after supratentorial neurosurgery. Neurology (2008) 71:665–9 10.1212/01.wnl.0000324624.52935.46 18725591

[59] Zachenhofer I Donat M Oberndorfer S Roessler K Perioperative levetiracetam for prevention of seizures in supratentorial brain tumor surgery. J Neurooncol (2011) 101:101–6 10.1007/s11060-010-0298-2 20526797

[60] Hildebrand J Lecaille C Perennes J Delattre JY Epileptic seizures during follow-up of patients treated for primary brain tumors. Neurology (2005) 65:212–5 10.1212/01.wnl.0000168903.09277.8f 16043788

[61] Fountain N Should levetiracetam replace phenytoin for seizure prophylaxis after neurosurgery? Epilepsy Curr (2009) 9:71–2 10.1111/j.1535-7511.2009.01297.x 19471614 PMC2685883

[62] UCB X Keppra (levetiracetam) injection for intravenous use. In: Package Insert. Smyrna, GA: UCB (2011), p. 1–16

[63] Ng YT Hastriter EV Cardenas JF Khoury EM Chapman KE Intravenous levetiracetam in children with seizures: a prospective safety study. J Child Neurol (2010) 25:551–5 10.1177/0883073809348795 20413804

[64] Ramantani G Ikonomidou C Walter B Rating D Dinger J Levetiracetam: safety and efficacy in neonatal seizures. Eur J Paediatr Neurol (2011) 15:1–7 10.1016/j.ejpn.2010.10.003 21094062

[65] Reiter PD Huff AD Knupp KG Valuck RJ Intravenous levetiracetam in the management of acute seizures in children. Pediatr Neurol (2010) 43:117–21 10.1016/j.pediatrneurol.2010.03.017 20610122

[66] Kirmani BF Crisp ED Kayani S Rajab H Role of intravenous levetiracetam in acute seizure management of children. Pediatr Neurol (2009) 41:37–9 10.1016/j.pediatrneurol.2009.02.016 19520272

[67] Abend NS Gutierrez-Colina AM Monk HM Dlugos DJ Clancy RR Levetiracetam for treatment of neonatal seizures. J Child Neurol (2011) 26:465–70 10.1177/0883073810384263 21233461 PMC3082578

[68] Khan O Chang E Cipriani C Wright C Crisp E Kirmani B Use of intravenous levetiracetam for management of acute seizures in neonates. Pediatr Neurol (2011) 44:265–9 10.1016/j.pediatrneurol.2010.11.005 21397167

[69] Gallentine WB Hunnicutt AS Husain AM Levetiracetam in children with refractory status epilepticus. Epilepsy Behav (2009) 14:215–8 10.1016/j.yebeh.2008.09.028 18926926

[70] Goraya JS Khurana DS Valencia I Melvin JJ Cruz M Legido A Intravenous levetiracetam in children with epilepsy. Pediatr Neurol (2008) 38:177–80 10.1016/j.pediatrneurol.2007.11.003 18279751

[71] Abend NS Monk HM Licht DJ Dlugos DJ Intravenous levetiracetam in critically ill children with status epilepticus or acute repetitive seizures. Pediatr Crit Care Med (2009) 10:505–10 10.1097/PCC.0b013e3181a0e1cf 19325512 PMC2946960

[72] Depositario-Cabacar DT Peters JM Pong AW Roth J Rotenberg A Riviello JJ Jr High-dose intravenous levetiracetam for acute seizure exacerbation in children with intractable epilepsy. Epilepsia (2010) 51:1319–22 10.1111/j.1528-1167.2010.02519.x 20163437

[73] Cilio MR Bianchi R Balestri M Onofri A Giovannini S Di Capua M Intravenous levetiracetam terminates refractory status epilepticus in two patients with migrating partial seizures in infancy. Epilepsy Res (2009) 86:66–71 10.1016/j.eplepsyres.2009.05.004 19520548

[74] Haberlandt E Sigl SB Scholl-Buergi S Karall D Rauchenzauner M Rostasy K Levetiracetam in the treatment of two children with myoclonic status epilepticus. Eur J Paediatr Neurol (2009) 13:546–9 10.1016/j.ejpn.2008.09.006 19010072

[75] Wheless JW Clarke D Hovinga CA Ellis M Durmeier M McGregor A Rapid infusion of a loading dose of intravenous levetiracetam with minimal dilution: a safety study. J Child Neurol (2009) 24:946–51 10.1177/0883073808331351 19264738

[76] Bilo L Meo R Striano S De Leva M Buongiovanni A Di Nocera P Use of levetiracetam plasma levels monitoring in the management of patients with epilepsy [abstract]. Epilepsia (2004) 45(Suppl 7):118

[77] May TW Rambeck B Jurgens U Serum concentrations of levetiracetam in epileptic patients: the influence of dose and co-medication. Ther Drug Monit (2003) 25:690–9 10.1097/00007691-200312000-00007 14639055

[78] Ben-Menachem E Falter U Efficacy and tolerability of levetiracetam 3000 mg/d in patients with refractory partial seizures: a multicenter, double-blind, responder-selected study evaluating monotherapy. European Levetiracetam Study Group. Epilepsia (2000) 41:1276–83 10.1111/j.1528-1157.2000.tb04605.x 11051122

[79] Betts T Waegemans T Crawford P A multicentre, double-blind, randomized, parallel group study to evaluate the tolerability and efficacy of two oral doses of levetiracetam, 2000 mg daily and 4000 mg daily, without titration in patients with refractory epilepsy. Seizure (2000) 9:80–7 10.1053/seiz.2000.0380 10845730

[80] Cereghino JJ Biton V Abou-Khalil B Dreifuss F Gauer LJ Leppik I Levetiracetam for partial seizures: results of a double-blind, randomized clinical trial. Neurology (2000) 55:236–42 10.1212/WNL.55.2.236 10908898

[81] Shorvon SD Lowenthal A Janz D Bielen E Loiseau P Multicenter double-blind, randomized, placebo-controlled trial of levetiracetam as add-on therapy in patients with refractory partial seizures. European Levetiracetam Study Group. Epilepsia (2000) 41:1179–86 10.1111/j.1528-1157.2000.tb00323.x 10999557

[82] Perucca E Gidal BE Baltes E Effects of antiepileptic comedication on levetiracetam pharmacokinetics: a pooled analysis of data from randomized adjunctive therapy trials. Epilepsy Res (2003) 53:47–56 10.1016/S0920-1211(02)00250-4 12576167

[83] Folland C Moriarty GL Clinical experience of levetiracetam (LEV) in refractory adult epilepsy patients. In: Annual Meeting of the American Epilepsy Society. Seattle, WA: Blackwell Publishing, Inc. (2002). 192 p.

[84] Mushtaq R Wannamaker BB Levetiracetam blood levels have utility in clinical management of epilepsy. In: Annual Meeting of the American Epilepsy Society; Seattle, WA: Blackwell Publishing, Inc (2002). 107 p.

[85] Giroux PC Salas-Prato M Theoret Y Carmant L Levetiracetam in children with refractory epilepsy: lack of correlation between plasma concentration and efficacy. Seizure (2009) 18:559–63 10.1016/j.seizure.2009.05.007 19546014

[86] Lindholm D Levetiracetam levels correlating with successful treatment of epilepsy, headaches, cognitive effects, and adverse reactions in the pediatric age group. Epilepsia (2002) 43:60

